# Dual-Energy CT as a Well-Established CT Modality to Reduce Contrast Media Amount: A Systematic Review from the Computed Tomography Subspecialty Section of the Italian Society of Radiology

**DOI:** 10.3390/jcm13216345

**Published:** 2024-10-23

**Authors:** Susanna Guerrini, Matteo Zanoni, Cristian Sica, Giulio Bagnacci, Nicoletta Mancianti, Giuseppe Galzerano, Guido Garosi, Laura Maria Cacioppa, Michaela Cellina, Giulia A. Zamboni, Giuseppe Minetti, Chiara Floridi, Maria Antonietta Mazzei

**Affiliations:** 1 Unit of Diagnostic Imaging, Department of Medical Sciences, University of Siena, Azienda Ospedaliero-Universitaria Senese, 53100 Siena, Italy; guerrinisus@gmail.com; 2 Italian Society of Medical and Interventional Radiology (SIRM), Italian College of Computed Tomography, Italian Society of Medical and Interventional Radiology, 20122 Milano, Italy; giuliobagnacci@gmail.com (G.B.); michaela.cellina@asst-fbf-sacco.it (M.C.); giulia.zamboni@univr.it (G.A.Z.); dott.minetti@gmail.com (G.M.); chiara.floridi@gmail.com (C.F.); mamazzei@gmail.com (M.A.M.); 3 Unit of Diagnostic Imaging, Department of Medical, Surgical and Neuro Sciences and of Radiological Sciences, University of Siena, Azienda Ospedaliero-Universitaria Senese, 53100 Siena, Italy; matteo.zanoni.91@gmail.com (M.Z.); c.sica1@student.unisi.it (C.S.); 4 Unit of Nephrology, Dialysis and Transplantation, Department of Emergency and Transplantation, Azienda Ospedaliero-Universitaria Senese, 53100 Siena, Italy; mancianti25121988@gmail.com (N.M.); g.garosi@ao-siena.toscana.it (G.G.); 5 Unit of Vascular Surgery, Department of Heart, Thorax and Vessels, University of Siena, Azienda Ospedaliero-Universitaria Senese, 53100 Siena, Italy; galzerano.giuseppe@gmail.com; 6 Department of Clinical, Special and Dental Sciences, University Politecnica Delle Marche, 60126 Ancona, Italy; 7 Radiology Department, Fatebenefratelli Hospital, ASST Fatebenefratelli Sacco, Principessa Clotilde 3, 20121 Milan, Italy; 8 Institute of Radiology, Department of Diagnostics and Public Health, Policlinico GB Rossi, University of Verona, 37134 Verona, Italy; 9 Radiology Unit, Ospedale Santo Spirito, ASL AL Casale Monferrato, 15121 Alessandria, Italy

**Keywords:** dual-energy CT, single-energy CT, contrast media, contrast-induced nephropathy prevention, contrast volume, iodinated contrast shortage

## Abstract

**Background:** Our study aims to provide an overview of existing evidence regarding the image quality of dual-energy CT (DECT) employing reduced contrast media (CM) volumes, in comparison to single-energy CT (SECT) with standard CM loads. The advantages, indications, and possible applications of DECT were investigated from the perspective of providing better patient care, minimizing CM volume and managing CM shortage. **Methods**: In this systematic review (PRISMA methodology), PubMed and WOS were searched from January 2010 to January 2023 by two independent reviewers. The scan and CM characteristics, radiation dose, and results of quantitative (contrast to noise ratio, CNR, and signal to noise ratio, SNR) and qualitative assessment of image quality were collected. Sixty non-duplicated records eligible for full-text screening were examined. **Results**: Finally, 22 articles (1818 patients) were included. The average CM reduction with DECT ranged between 43.4 ± 11%. Despite the wide variability in CT scan protocols, no differences were found in radiation doses between DECT and SECT. **Conclusions:** DECT scanners allow the employment of lower CM volumes with equal or better image quality evaluated by quantitative and qualitative analyses and similar dose radiation compared to SECT. Using image reconstructions at low monochromatic energy levels, DECT increases iodine conspicuity and attenuation contributing to CM containment measures.

## 1. Introduction

Contrast-induced acute kidney injury (CI-AKI) is defined as iatrogenic kidney damage occurring after intravascular administration of contrast media (CM) for diagnostic or therapeutic purposes [1]. This clinical entity, previously named as contrast-induced nephropathy (CIN), refers to acute renal injuries occurring within 48 h from intravenous CM administration and after the exclusion of other nephrotoxic factors [2]. Nevertheless, CIN includes several overlapping acute renal injuries, unrelated to CM injection [2,3,4]. According to the Kidney Global Disease Outcomes Improvement Clinic (KDIGO) practical guidelines, CI-AKI occurs when serum creatinine (Cr) increases by at least 0.3 mg/dL from the baseline value within 48–72 h after intravenous CM administration, or when the increase is greater than 1.5 times compared to the reference value, within seven days after exposure to CM [5], as reported in Table 1.

CI-AKI is mostly transient. After contrast exposure, Cr usually returns to baseline within 14 days. Clinical symptoms of AKI are relatively mild or absent. However, AKI is associated with a prolonged hospital stay and a higher incidence of renal and cardiovascular adverse events [6,7]. Three pathophysiological mechanisms underlying contrast renal damage have been proposed, including direct tubular toxicity, intra-renal vasoconstriction, and excessive production of reactive oxygen species (ROS) [8]. The incidence of AKI in the general population has been reported to range from 1% to 3% [9]. The broad range reported by clinical studies is due to differences in definition and risk factors. The identified risk factors for AKI are chronic renal failure, diabetes mellitus, acute myocardial infarction, shock, and a high volume of CM administered [10,11]. The risk of AKI and its clinical course are similar after intra-arterial and intravenous CM administration [12]. Several studies in the literature suggest a dose-dependent risk of AKI within a contrast class when both the iodine concentration and the CM volume have been considered [13]. Furthermore, current evidence suggests that non-ionic iso-osmolar CM delivers the lowest risk of CI-AKI [14].

In the current perspective to reduce complications related to contrast nephropathy, the advent of the COVID-19 pandemic has emphasized the need, already clinically evident, to reduce the volume of CM administered during diagnostic and therapeutic procedures [15]. The COVID-19 pandemic has also reduced the availability of CM secondary to a massive manufacturing shutdown with great impact on patient care. A need has thus emerged to implement a series of maneuvers aimed at reducing the amount of the administered CM, involving the minimizing of the indications and the preference for diagnostic methods allowing the reduction in the amount of CM administered [16,17].

Single-energy CT (SECT) uses a single beam of polychromatic X-rays (70 to 140 kVp) emitted from a single source and received by a single detector.

The image obtained from this process depends on the alterations in the photon attenuation of the many materials that make up the human body. However, components that have different fundamental conformations (soft tissue, fat, air, calcium) can be characterized by the same CT numbers, which makes it impossible to differentiate and organize different tissue types, and this therefore represents a limitation of SECT. In dual-energy CT (DECT), on the other hand, the attenuation of tissues can be manipulated by changing the energy levels of photons, which is the fundamental factor in image composition.

In other words, DECT has been introduced as a first-generation spectral CT system, represented by dual-source or dual-layer technology, overcoming the tissue characterization limitations encountered with SECT. DECT also laid the foundation for the development of the recent photon-counting CT technology, paving the way for the extension of CT towards multi-energy CT imaging.

In this scenario, the introduction of dual-energy CT (DECT) scanners allows for lower volumes of CM with equal or better diagnostic information in comparison to single-energy CT (SECT) [17,18,19]. Among the mentioned risk factors for CI-AKI, CM alone can be effectively modified, thereby also impacting CM scarcity. Particularly, in patients with impaired renal function undergoing contrast-enhanced CT, the risk of CI-AKI has proven to be related to the volume of the iodine load [20]. Dual-energy CT (DECT) allows the use of image reconstruction at different monochromatic energy levels, increasing iodine conspicuity throughout low-energy datasets of images, with a higher attenuation than single-energy CT (SECT) and without compromising image quality [21,22,23,24].

The main purpose of this systematic review is to provide an overview of the existing evidence regarding the image quality of DECT examination with a reduced CM volume, in comparison to SECT examination with a standard CM load. We also described the indications, the advantages, and the possible applications of DECT, with the aim of improving patient care while minimizing the CM volume administered, thus contributing to CM containment measures.

## 2. Materials and Methods

This systematic review was performed in accordance with the Preferred Reporting Items for Systematic Reviews and Meta-Analyses (PRISMA) [25]. Due to the study design, institutional review board approval was not required and written informed consent was waived.

### 2.1. Literature Search Strategy and Study Selection

A literature search was conducted on 15 December 2023. The Medline (via PubMed) and Web of Science databases were searched by two independent reviewers for relevant studies, published from January 2010 to January 2023, comparing the image quality of DECT examination using an iodine low-dose strategy with standard iodine load SECT examination. The references for the collected articles were cross-checked for additional relevant studies.

The PubMed and Web of Science databases were searched using the following combinations of keywords: (1) “iodine load” OR “iodine” OR “iodinated contrast media” OR “iodinated contrast agent” OR “contrast media” OR “contrast agent” AND (2) “reduction” OR “low dose” AND (3) “dual-energy” OR “dual-energy CT” OR “dual-energy computed tomography” OR “DECT” OR “spectral CT”. Clinical questions regarding iodine contrast media reduction using DECT were developed in the population, intervention, comparator, and outcome (PICO) format.

### 2.2. Inclusion and Exclusion Criteria

Inclusion and exclusion criteria are reported in Table 2.

Eligibility screening was first conducted by screening the title and abstract for matching the inclusion criteria. Then, a full-text screening for eligibility was performed. Disagreements were resolved by consensus.

### 2.3. Literature Search

An initial search using the word combination reported above yielded 4635 PubMed articles and 66,735 articles from Web of Science. After application of the inclusion/exclusion criteria, our literature search yielded 950 citations.

**Figure 1:**
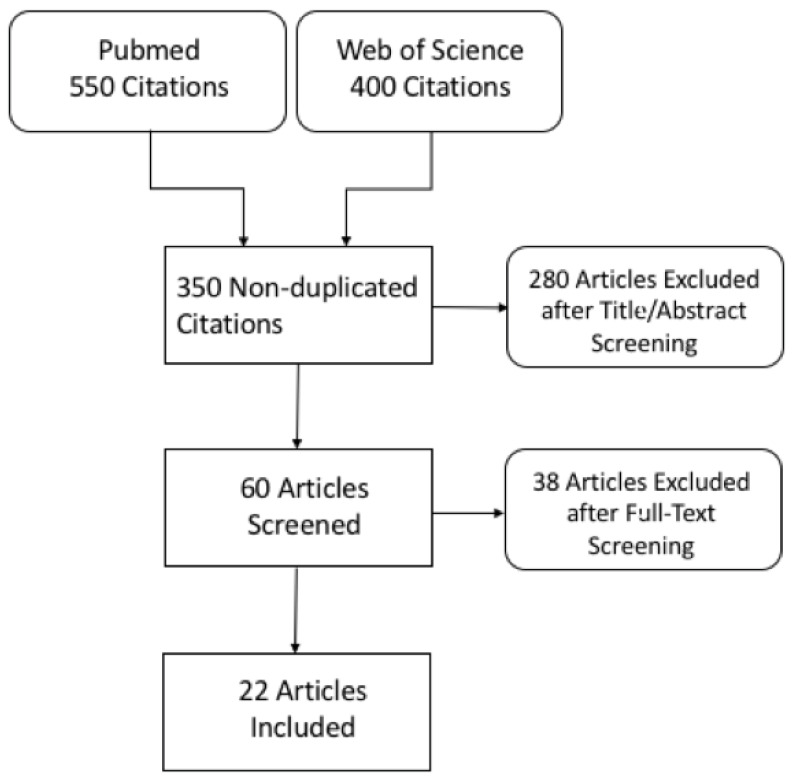
Flowchart of literature search and study selection.

Following title and abstract screening, 60 non-duplicated records were eligible for full-text screening and were examined in detail. Finally, 22 articles were included in this systematic review. In four studies, patients underwent both DECT and SECT examinations (in-patient comparison). A flow diagram showing the literature search and selection of articles for review is shown in Figure 1.

### 2.4. Data Extraction and Quality Assessment

For the eligible studies that met the inclusion and exclusion criteria, data were extracted independently by two reviewers, and discrepancies were resolved by consensus. The selected studies were divided into several groups according to the main purpose of DECT examination (coronary arteries, pulmonary vessels, abdomen, and aorta). If more than one body district was examined (for example, aorta and liver or coronary artery and aorta), data about all the considered structures were collected.

The following characteristics were collected and reviewed: author and year of publication, number of patients, age, sex, body mass index (BMI), type of DECT scanner, scan parameters for DECT and SECT, CM protocol, radiation dose (CTDIvol and/or DLP), and results of quantitative (contrast to noise ratio, CNR, and signal to noise ratio, SNR) and qualitative assessments of image quality (evaluated through a 3- to 10-point Likert scale).

The methodological quality of available research was evaluated by a checklist based on the Quality Assessment of Diagnostic Accuracy Studies-2 (QUADAS-2) [26].

### 2.5. Outcome Measures

The primary outcome was to compare the image quality of DECT imaging using an iodine low-dose strategy with standard iodine load SECT examinations.

As a secondary outcome, we evaluated and compared the radiation doses of DECT and SECT examinations.

### 2.6. Study Risk of Bias Assessment

This study could be subject to selection bias in the case of a lack of adequate randomization of the studies searched in the literature (adequate methods include a random number table, computer-generated randomization, random-element-free minimization). A second bias could also refer to the incorrect consideration of baseline characteristics (considering whether systematic differences in the baseline characteristics of studies and contrast agent exposure factors were observed between different groups, whether important differences were observed, whether analyses controlled for these differences). Another possible bias relates to attrition bias in the case of incomplete outcome data (considering whether incomplete outcome data were treated appropriately, including systematic differences in attrition between groups [differential attrition SECT vs. DECT]).

We studied the overall quality rating of low risk. The approach to select studies from the literature uses appropriate means to prevent bias, measure outcomes, and analyze and report the results.

## 3. Results

### 3.1. Literature Search Results

In total, 4 out of 22 studies were focused on CT coronary angiography (CTCA) [27,28,29,30] and 2/22 on CT pulmonary angiography (CTPA) [31,32], while 1/22 assessed both pulmonary arteries and veins [33]. In total, 9 of the 22 studies were related to abdominal CT [21,34,35,36,37,38,39,40,41] while thoracic and/or abdominal aorta and its branches were evaluated in 14/22 articles, of whom 6 studies included thoracic and/or abdominal aorta alone [42,43,44,45,46,47]. In one study, vascular evaluation was associated with coronary arteries [28], in one study with pulmonary vessels [33], and in six records with the abdomen [21,34,36,37,39,41].

### 3.2. Characteristics of the Included Studies’ Population

A total of 1818 patients (1148 males) were included in our systematic review. The mean age was the lowest for Li et al. (42.25 ± 13.22 years), while the highest mean age (77 ± 10.25 years) was reported by Patino et al. [38,46]. Of all of the included patients, 1127 and 743 patients underwent DECT and SECT examination, respectively. In all of the recorded studies, no significant differences were found in terms of sex, age, and BMI distribution between the DECT and SECT groups.

### 3.3. Characteristics of CT Scans and CM Protocols

In 13/22 studies, the results were obtained using an SSrs 64-MDCT scanner, in 3/22 studies an SSdl 128-MDCT scanner was used, and in 2/22 studies a DS 128-MDCT scanner was used. In the remaining 4/22 studies, different scanners were used for SECT and DECT. In total in the studies, the reduction in the administered CM with DECT ranged between 23% and 60%, with an average CM reduction of 43.4 ± 11%, and 14/22 studies showed a recorded iodine reduction ≥ 50%.

The baseline characteristics of the study populations, CT examinations, and CM protocols are shown in Table 3.

### 3.4. Quantitative and Qualitative Image Assessment

The most used virtual monoenergetic images (VMI) reconstructions were 40, 50, 60, and 70 keV, regardless of the anatomical district being examined, with maximum CM attenuation at 40 keV. Concerning quantitative analysis, CNR was analyzed in 21/22 studies, while SNR was analyzed in 9/22 of the recorded studies. The qualitative assessment consisted of subjective scales to evaluate the overall image quality and performed through a 3-point scale in 1 study, a 4-point scale in 6/22 studies, a 5-point scale in 13/22 studies, a 6-point scale in 1 study, and a 10-point scale in the remaining study. In 12/22 studies, a subjective scale to evaluate image noise was also adopted. The results of quantitative and qualitative image assessment are summarized in Table 4, Table 5 and Table 6.

Although there was significant variation in CT scan protocols across the studies included in this review, the findings indicate that, for the most part, there is no significant difference in the radiation doses between DECT and SECT examinations. In particular, the radiation doses were significantly higher for DECT in four studies [31,33,46,47], two of which assessed the abdominal aorta [46,47] and two pulmonary arteries [31,33]. On the contrary, the radiation dose was reported to be lower in the DECT group in four articles [38,41,44,45], two of which focused on abdominal CT [38,41] and two on aorta examination [44,45].

## 4. Discussion

CI-AKI is reported as the third most common cause of acute kidney injury in hospitalized patients resulting in a significant increase in morbidity and mortality, especially in fragile patients [34]. CI-AKI frequency is particularly high in the diabetic patient population. According to the International Diabetes Federation (IDF), the prevalence of diabetes in adults is predicted to rise to 10.4% of the general population in the next 10 years, leading to an increase in diabetic patients requiring computed tomography with CM administration [48,49]. The KDIGO Clinical Practice Guidelines for AKI state that no definitive treatment exists for established AKI, claiming that prevention is currently the best option [5]. Intravenous hydration has been reported as the cornerstone in AKI prevention; however, organizational barriers and the increasing need for contrast imaging examinations are an obstacle to the implementation of hydration strategies in clinical practice [50]. Furthermore, the limited availability of CM resources and the expectation of a further reduction in the near future make an immediate solution to the problem necessary [51]. A reduction in the volumetric dose of CM could represent the focus in AKI prevention being the most modifiable and customizable factor [52,53]. Recent technical development may prove that DECT can be a valid option for reducing CM administration while maintaining the same diagnostic quality [54]. This advantage is particularly valuable for the elderly population and patients with impaired renal function or comorbidities such as diabetes, who are projected to increase in prevalence in the foreseeable future. Furthermore, this approach offers economic benefits and savings, allowing a 50% reduction in the CM dose for each individual examination, regardless of the clinical indication.

**Figure 2:**
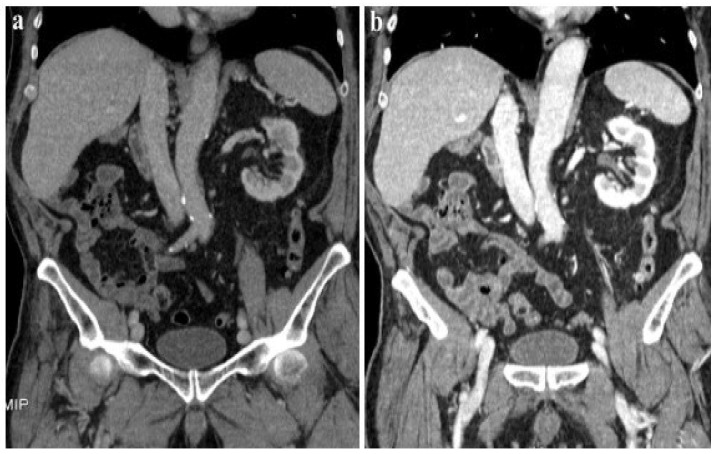
A 120 kVp SECT performed with a standard iodine dose (**a**) compared to VMI at 40 KeV (DECT) performed with a reduced iodine dose (**b**) in a 80-year-old patient with impaired renal function. VMI (**b**) allows a significative reduction in CM administration and improved image quality compared to 120 kVp SECT (**a**).

According to our results, no significant differences were found in vascular and parenchymal enhancement in DECT imaging with a reduced CM protocol as compared to conventional SECT with a standard weight-based CM protocol (Figure 2).

Despite the added value of DECT having been widely recognized, the improved image quality and the possibility to reduce CM are also affected by the different DECT technologies [55]. The commercially available DECT platforms currently include dual-source, single-source with fast kV-switching, single-source split-filter, and dual-layer technologies. The improved image quality and cost-effectiveness of some DECT scanners have promoted their prevalence in routine practice [56]. The choice of DECT scanners should also be adapted to patient characteristics, clinical settings, specific applications, and financial means [55]. Temporal resolution, patient size, motion, and beam-hardening artifacts are the most important factors to be considered for accurate patient selection [55,57].

**Figure 3:**
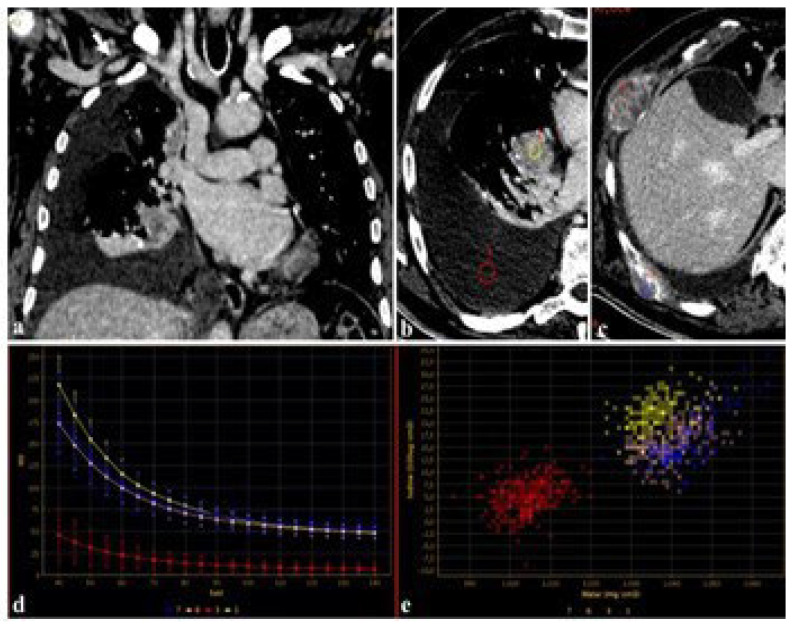
An 83-year-old male candidate for transcatheter aortic valve implantation (TAVI) submitted to DECT examination. The study of the thoracoabdominal aorta (**a**), performed with 85 mL of contrast media for the evaluation of peripheral accesses ((**a**), arrows), revealed a pulmonary right hilar cancer (**b**) with osteolytic bone metastases (**c**). After drawing circular regions of interest (ROIs) within right hilar mass, pleural effusion (**b**), and bone metastases (**c**), the attenuation was measured at the given energy level, demonstrating the absence of disease in the pleural effusion (**b**,**d**,**e**), further confirmed by spectral iodine maps ((**d**), spectral curve, (**e**), scatterplot GSI).

Furthermore, using DECT, the attenuation of iodine for a given region of interest (ROI) can be represented as a spectral curve representing the change in attenuation values and iodine uptake in different ROIs [35]. These spectral curves can be used in the evaluation of primary and secondary lesions, regardless of the amount of CM employed (Figure 3).

**Figure 4:**
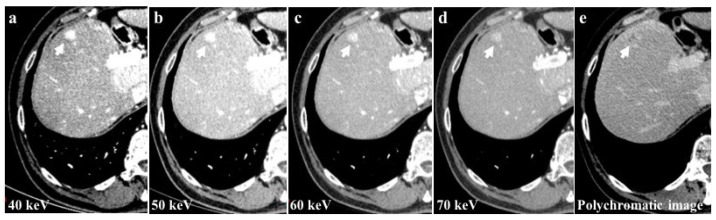
The 40 keV images of a 56-year-old patient with hepatocellular carcinoma (HCC) of the 8th liver segment (**a**) showed a higher contrast media attenuation and the highest difference between the background parenchymal and the lesion enhancement (arrows) compared to 50 keV (**b**), 60 keV (**c**), and 70 keV (**d**) image reconstructions. Monochromatic energy levels (40–70 keV) also increase the detectability of small HCCs and cirrhotic liver disease when compared to conventional polychromatic imaging (**e**).

Among the examined study examinations, the 40 keV images exhibit the highest CM attenuation when compared to the 50 keV, 60 keV, and 70 keV images. Moreover, the 40 keV images demonstrate the most pronounced enhancement of vascular structures and parenchyma (Figure 4).

**Figure 5:**
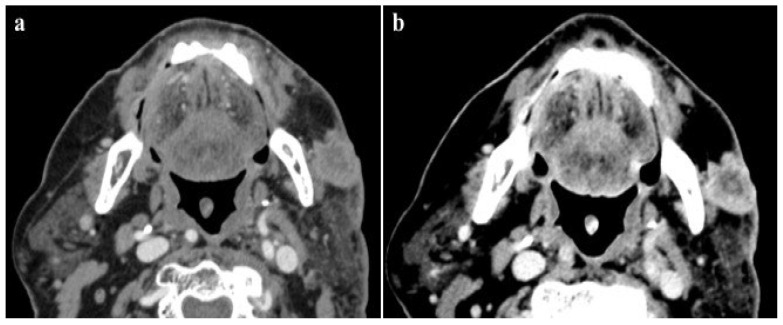
A 95-year-old male patient with low glomerular filtrate rate (GFR 30 mL/min), submitted to a DECT examination for loco-regional neoplasm staging after the injection of 60 mL of contrast medium. The detection of the lesion, as well as the evaluation and definition of skin infiltration of this enhancing tumor, were better evaluated by the low-energy image (40 keV) (**a**) when compared to the corresponding polychromatic image (**b**), with a consistently reduced amount of contrast agent.

This evidence offers the possibility to reduce the amount of CM administered by 50% while maintaining the same diagnostic performance as the SECT acquisition with the standard amount of CM (Figure 5).

Similar results lead to a large impact on the ability to purchase and employ CM, in accordance with the most recent ACR Committee on Drugs and Contrast Media recommendations, with the aim to provide high-quality patient care during CM shortage times [58,59].

### 4.1. CT Coronary Angiography (CTCA)

In Raju et al. [27], Oda et al. [28], Rotzinger et al. [29], and Carrascosa et al. [30], rates of iodine load reduction ranged between 40% and 56%. The preferred monoenergetic level for VMIs were 60 keV in two studies [27,30], 55 keV in one [29] and 50 keV in the remaining study [28]. CNR was available in all studies and resulted significantly higher in most of cases. Oda et al. [28] and Rotzinger et al. [29] reported the highest values of CNR for DECT. The highest value of SNR, which was assessed only by two articles, was found in the SECT group of Carrascosa et al. [30] and was significantly higher than the mean SNR in the DECT group. Despite the different evaluation scales used for qualitative image quality assessment, in all the four studies, DECT and SECT showed comparable subjective quality scores, except for Raju et al. [27], where the radiologist with 18 years’ experience gave a significantly higher score for SECT images. Nevertheless, scores ranged from good to excellent in all the considered studies.

In the coronary artery study, the preferred single-energy level for VMI was found to be between 50 and 60 keV for the same 40–56% reduction in MDC compared to SECT. The highest SNR value was found in the SECT group.

### 4.2. CT Pulmonary Angiography (CTPA) and CT for Pulmonary Vessels

Concerning Dong et al. [31] and Yuan et al. [32], studies focused on CTPA, both adopted a 50% lower CM protocol with DECT and a VMIs level of 50 keV and 70 keV, respectively. Both CTPA studies showed significantly better CNR and SNR values for VMIs at an energy level close to 50 keV with the best results achieved by Dong et al. [31]. Furthermore, in the latter, the 70 keV VMIs quality was not inferior to SECT images in statistical analysis. The qualitative assessment evaluated on a 5-point scale was diagnostic in both studies, with a subjective preference for SECT images in Yuan et al. [32] and for VMIs in Dong et al. [31]. Delesalle et al. [33]. evaluated the image quality for pulmonary vessels (pulmonary artery and veins) in the arterial phase, using DECT with 60 keV VMIs and a 30% reduction in iodine load. At the qualitative assessment on a 3-point scale, subject noise was scored as absent or acceptable in most cases for both DECT and SECT groups, without any statistically significant differences [33].

### 4.3. Abdomen

In the nine studies focused on abdominal CTs included in the present review [21,34,35,36,37,38,39,40,41], the iodine dose reduction ranged from 30% to 50% of the standard iodine load.

#### 4.3.1. Liver, Portal Vein and Pancreas

Two articles evaluated the absolute liver CNR [34,39], one of which also included pancreas CNR evaluation [34]. Clark et al. [34] reported a slightly worse, but not significant, CNR in 52 keV VMIs than SECT for both liver and pancreas, while in Lv et al. [39] the CNR for the liver at the portal venous phase (PVP) was significantly higher in 40 keV VMIs. Moreover, three articles assessed CNR between liver and hyperenhancing focal hepatic lesions [36], hepatic tumors [41] and typical hemangiomas [38] in 40, 40, and 52 keV VMIs, respectively. The CNR was significantly higher in all three studies, especially in the arterial phase. The SNR in the liver (liverSNR) was assessed by Kim et al. [36] and Lennartz et al. [37] and it was found to be lower at 40 keV VMIs in both the arterial phase (AP) and PVP. An overall direct proportionality between the liverSNR and the energy level in the late arterial phase was detected. Conversely, the liverSNR in PVP was lower in VMIs at higher energy levels, as reported by Kim et al. [36]. No significant difference in the pancreas SNR was found between 40 keV VMIs and SECT images.

The CNR in the portal vein and its branches were systematically assessed for intra- and extra-hepatic vessels by Han et al. [35] and Ma et al. [40], resulting in significantly higher values for the intrahepatic portal vein in 60 keV VMIs compared to SECT images in Ma et al. [40]. The CNR of portal veins was also assessed by Lv et al. [39] and found to be significantly higher in 40 keV VMIs at PVP. The SNR in portal veins was assessed by Kim et al. [36], reporting a significantly higher value in the DECT group (40 keV VMIs at PVP) in comparison to the control group (SECT). In most of the studies, subjective analysis did not show any statistical difference in terms of image quality and diagnostic acceptability, as both DECT and SECT scan protocols provided good or excellent quality and similar noise. Lennartz et al. [37] was the only series reporting a significant subjective preference for SECT images, with a median score of 3/4 (i.e., “proper overall assessability”) for 40 keV VMIs and 4/4 (i.e., excellent overall assessability) for 120 kVp images [37]. Nagayama et al. [41], who used a 5-point scale score, instead reported a significantly superior overall quality score in 40, 45, and 50 keV VMIs compared to 120 kVp. Despite Li et al. [38] finding no difference in image quality score, 52 keV showed a better lesion conspicuity than SECT.

#### 4.3.2. Kidneys and Urinary Tract

Two of all of the included studies focused on kidneys [19,35], one of which also evaluated the urinary tract [21]. In both articles, the DECT groups underwent a 50% lower CM protocol. In Shuman et al. [21], a 50 keV VMI was evaluated; the renal parenchyma at 90 s and all of the urinary tract at the delayed phase showed a better CNR for DECT. Furthermore, at qualitative assessment, the subjective enhancement and image noise were similar between DECT and SECT patient groups. In Lennartz et al. [37], a 40 keV VMI was analyzed, and no differences were found in terms of the SNR between DECT and SECT imaging.

For the study of the abdomen (liver, portal vein, pancreas, kidney, and urinary tract) with a reduction in MDC dose ranging from 30% to 50% of the standard iodine load compared to SECT, using VMI between 40 and 60 KeV, overall better or equal CNR and SNR values were obtained compared to SECT.

### 4.4. Aorta and Its Branches

Of all the articles included, 2 studies assessed ascending aorta [28,44], 2 descending aorta [33,44], 1 toraco-abdominal aorta [43], and 11 abdominal aorta and/or its branches [21,34,36,37,39,41,42,44,45,46,47].

Over a total of 14 studies, in 6 studies the iodine dose was reduced by at least 50%, with a maximum reduction rate of up to 70% in Carrascosa et al. [43]. Quantitative imaging evaluation was clearly in favor of DECT in 8 out of 14 studies, for both CNR and SNR, with the highest values reported at the energy levels of 40 and 50 keV in Carrascosa et al. [43]. The CNRs evaluated on VMIs were higher or equivalent to those of control groups along all aorta and its branches [43]. DECT was not inferior to SECT even though only 30% of the standard iodine dose was administered [43]. In addition, Liu et al. [45], who considered obese patients alone, reported significantly higher values of CNR for the DECT group with a 23% reduced dose of CM, although 65 keV was set as the energetic level. Qualitative assessment was similar between case and control groups in almost all studies, both regarding image quality and noise, despite a significant drop in image quality being reported by Carrascosa et al. [43] in the DECT group receiving 30% of the standard CM dose.

In the study of the aorta and its branches, the iodine dose can be reduced from a minimum of 50% to a maximum of 70%, with a significant improvement in image quality in favor of DECT compared to SECT, with energy levels between 40 and 50 keV.

### 4.5. Dose Exposure Considerations

**Figure 6:**
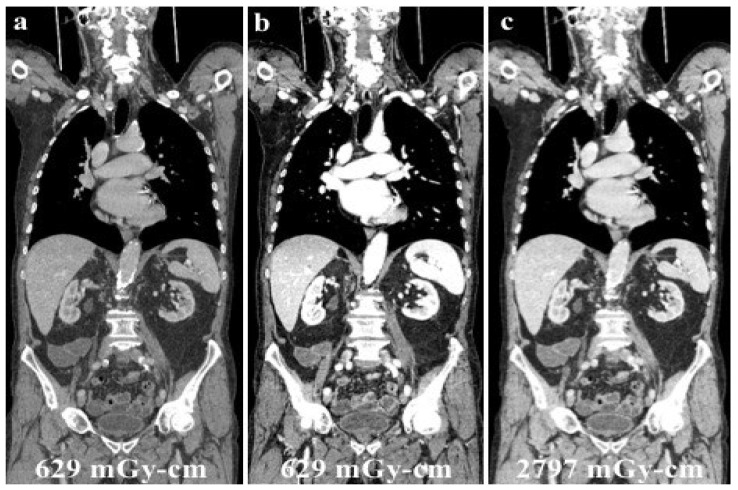
A young male patient receiving chemotherapy for a primary mediastinal large B-cell lymphoma submitted to routine DECT follow-up after 70 mL of contrast media administration (**a**), and evaluated at low energy levels (40 keV, (**b**)) to reduce the iodine load and maintain a high diagnostic accuracy. Low energy levels (**b**) were as accurate as SECT (**c**) in confirming no recurrence of disease with considerable advantages in the patient’s dose exposure (total DLP: (**a**,**b**), 629.18 mGy-cm; (**c**), 2797.25 mGy-cm).

Since its use in clinical practice, the issue of patient dose exposure with DECT has been the focus of several studies in the literature. Dual-energy CT has been mistakenly associated with exposure to a “double” dose of ionizing radiation, only because of the word “double” in its name. However, many series reported the feasibility of DECT without increasing the radiation dose exposure of the patient, as illustrated in the case shown in Figure 6 [60,61].

In those studies, the normalization of image quality, signal-to-noise ratio, and dose-length product (DLP) were registered with the aim of ensuring the proper characterization of dose exposure [62,63].

According to Yu et al., the results of the present review show that 40 KeV images yield a similar or even better iodine CNR than that of the typical 120 kV images acquired at the same radiation dose [64]. Furthermore, John et al. [65] observed the lowest radiation doses with DECT in third-generation dual-source CT, demonstrating that DECT can be routinely used with decreased radiation dose exposure in abdominal imaging.

**Figure 7:**
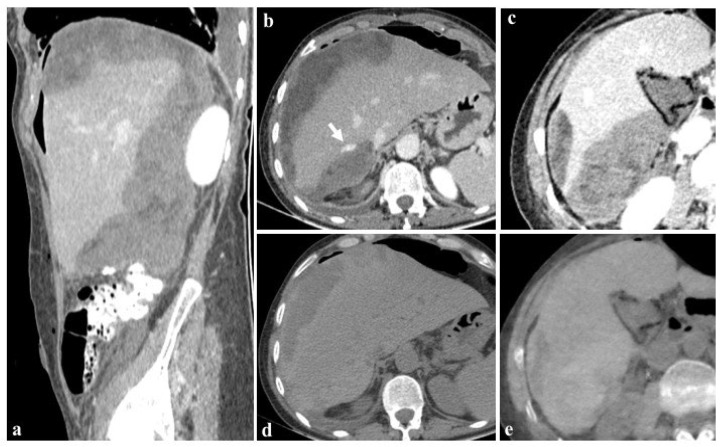
Images at 40 keV after 80 mL contrast medium (**a**–**c**) show the presence of an active bleeding liver ((**b**), arrow) and a diffuse subcapsular hematoma after cholecystectomy in a 76-year-old woman. The patient was rapidly referred to percutaneous angiographic treatment. True non-contrast imaging (**d**) may be replaced by virtual non-contrast reconstructions (**e**) in the evaluation of subcapsular hematoma avoiding unnecessary scans without contrast medium during post-embolization follow-up.

The utilization of iterative reconstruction algorithms is another significant factor contributing to dose reduction [66]. These algorithms facilitate the reduction in CT image noise while preserving the spatial resolution and contrast-to-noise ratio (CNR) [67]. Furthermore, the integration of virtual non-contrast (VNC) images, as in the case shown in Figure 7, obviates the necessity for true non-contrast (TNC) acquisitions, resulting in a substantial reduction in radiation dose exposure ranging from 32.9% [60] to 57% [68,69,70,71,72].

### 4.6. Study Limitations

Some limitations of this study should be discussed. The main limitation of the present review is the inability to perform a mathematical meta-analysis given the heterogeneity of the reported data in terms of the populations, DECT technology, scanning parameters, and CM injection protocols for each study. Furthermore, the evaluation of diagnostic accuracy in terms of sensitivity and specificity according to a reference standard is lacking since the data were mainly focused on quantitative and qualitative evaluation of image quality. Another limitation stems from the missing analysis of the possible use of different iterative reconstruction (IR) algorithms to reduce image noise due to the inhomogeneous populations.

## 5. Conclusions

DECT technology, which enables the generation of virtual monoenergetic images (VMIs) derived from different energy datasets (40–60 keV), offers the advantage of enhanced iodine contrast while minimizing the iodine load. A significant reduction in contrast-induced acute kidney injury (CI-AKI) following contrast media (CM) administration can be achieved with DECT employment, with comparable image quality measurements and scores to those obtained from standard 120 kVp SECT performed with standard iodine doses. Spectral CT is a constantly developing field and, although it has been in clinical practice for about 20 years, it has not stopped evolving both to overcome the limitations of DECT and in the development of new technologies such as spectral photon-counting CT technology.

## Figures and Tables

**Table 1:** Kidney Disease Improving Global Outcomes (KDIGO) 2012-based definition of the acute kidney injury (AKI) guidelines. Current criteria for diagnosing and staging AKI [5].

*KDIGO Clinical Practice Guidelines for Acute Kidney Injury (AKI)*	
Diagnostic Criteria for AKI	
Increase in serum creatinine by ≥0.3 mg/dL (26.5 μmol/L) within 48 h; or Increase in serum creatinine to ≥1.5 times baseline, known or presumed to have occurred in the past 7 days; or Urine volume < 0.5 mL/kg/h for 6 h	
AKI Staging	
*AKI stage I*	Increase by ≥0.3 mg/dL (26.5 μmol/L); or Increase to 1.5–1.9 times from baseline; or Urine volume < 0.5 mL/kg/h for 6–12 h
*AKI stage II*	Increase to 2.0–2.9 times from baseline; or Urine volume < 0.5 mL/kg/h for ≥ 12 h
*AKI stage III*	Increase ≥ 3 times from baseline; or Serum creatinine by ≥4 mg/dL (≥354 μmol/L); or Initiation of renal replacement therapy; or Decrease in eGFR to <35 mL/min/1.73 m^2^ in patients < 18 years; or Urine volume < 0.3 mL/kg/h for ≥24 h; or Anuria for ≥12 h

**Table 2:** Inclusion and exclusion criteria of the present systematic review.

*Inclusion and Exclusion Criteria*	
**Inclusion Criteria**	
*Study Design*	Observational case-control studies: - Prospective studies; - Retrospective studies.
*Population*	Case populations of patients submitted to contrast-enhanced DECT with a low-dose CM protocol, having as a control group patients submitted to contrast-enhanced SECT examination with a standard-dose CM protocol.
*Indicator*	Studies reporting results of qualitative and quantitative image quality assessment of both DECT and SECT examinations.
*Comparison*	Studies comparing DECT and SECT imaging.
**Exclusion Criteria**	
Studies that met any of the following criteria were excluded: - Abstracts, case reports, editorials letters, comments, and animal or phantom experiments; - Cases of duplicate or overlapping data with other studies; - English full-text article not available; - Studies including <20 patients; - Limited information available about CT scan protocols or regarding the dose of iodinated CM administered, for both groups; - Studies that did not report the results of qualitative and quantitative image quality assessment for both DECT and SECT examinations.	

DECT, dual-energy CT; SECT, single-energy CT.

**Table 3:** Summary of the baseline characteristics of the study populations, CT examination, and CM protocol.

	*Author* *(Year)* *n. of Patients*	*Purpose of CT* *Examination*	*n. of Patients* *(SECT* vs. *DECT)*	*CT Scanner* *(SECT* vs. *DECT)*	*CT Scan Protocol* *(SECT* vs. *DECT)*	*Contrast Protocol* *(SECT* vs. *DECT)*	*Dose* *(SECT* vs. *DECT)* *CTDIvol (mGy)* *DLP (mGy/cm)*
**1**	Raju et al. (2014) [27] *n* = 102 (57)	Coronary art.	53 vs. 49	SSrs 64-MDCT	100 (BMI < 30)/120 (BMI > 30) kVp, ATCM, NI 28, ASIR 40% vs. 80/140 kVp, 600 mA; 1.25 mm, 0.5 s, table feed/rotation 0.984 mm, prospective ECG-gating, test bolus	Iodixanol 320, 80 mL vs. 35 mL, 5.5 mL/s	164.79 (84.49) vs. 159.41 (46.73)
**2**	Carrascosa et al. (2015) [30] *n* = 36 (27)	Coronary art.	36 *	SSrs 64-MDCT	100 (BMI < 30)/120 (BMI > 30) kVp, mA based on BMI vs. 80/140 kVp, 600–640 mA, ASIR 40%; collim. 0.625 mm, 0.625/0.625 mm, 0.350 s, prospective ECG-gating	Iobitridol 350, BMIx0.9 mL, 4.5–5.0 mL/s vs. 50% of iodine dose, 4–5 mL/s	N/A
**3**	Oda et al. (2019) [28] *n* = 60 (35)	Coronary art., asc. aorta	30 vs. 30	SSdl 128-MDCT	120 kVp, ATCM, DRI 36, 0.67/0.33, 0.27 s, pitch 0.16, iDose 3 vs. Spectral level 0, retrospective ECG gating, bolus track (100 HU in asc. aorta +6 s)	Iopamidol 370, 280 mg/kg vs. 140 mg/kg, rate adjusted for 16 s IT	36.5 (8.2) vs. 33.3 (8.1)
**4**	Rotzinger et al. (2021) [29] *n* = 203 (114)	Coronary art.	103 vs. 100	SSdl 128-MDCT	120 kVp, ATCM (max 220 mA), collim. 0.625 mm, 0.9 mm, 0.27 s, iDose 3, retrospective ECG gating, bolus track. (130 HU in desc. Aorta)	Iomeprol 400, 1 mL/kg (max 90 mL), 5 mL/s vs. 0.5 mL/kg (max 45 mL), 2.5 mL/s	23 (13.5) vs. 21.6 (13.6); 430.7 (266.1) vs. 392.8 (251.7)
**5**	Yuan et al. (2012) [32] *n* = 94 (55)	Pulmonary art.	46 vs. 48	SSrs 64-MDCT	100 (BMI < 30)/120 (BMI < 30) kVp, ATCM, NI 28, ASIR 40%, vs. 80/140 kVp, 600 mA; 1.25 mm, 0.5 s, table feed/rotation 0.984 mm, test bolus	Ioversol 320 vs. 50%-diluted ioversol 320, CV = inj. rate * (preparation delay + scan time −6), 4–5 mL/s (based on BMI)	400.8 (208.7) vs. 412.5 (34.1)
**6**	Dong et al. (2013) [31] *n* = 86 (46)	Pulmonary art.	41 vs. 45	SSrs 64-MDCT	120 kVp, 300 mA, ASIR 30% vs. 80/140 kVp, ATCM (550 mA); 1.25 mm, 0.5 s, pitch 1.375:1, bolus track. (peak in main pulm. art.)	Iopamidol 370, 50 mL vs. 20 mL, 5 mL/s	7.06 (1.06) vs. 12.72 †; 195.1 (35.4) vs. 337.5 (29.9) †
**7**	Delesalle et al. (2013) [33] *n* = 110 (78)	Pulmonary art. and v., desc. aorta	30 vs. 80	DS 128-MDCT	120 kVp, 90 mA, 0.28 s, pitch 1.5 vs. 80/140 kVp, ATCM (250/149 mA), 0.33 s, pitch 1, FBP; collim. 2 × 0.6 mm, 1.0 mm, bolus track. (100 HU in asc. Aorta)	Iohexol 350, 90 mL vs. iohexol 170, 120 mL, 4 mL/s	166.13 (45.46) vs. 272.3 (59.24) ‡
**8**	Clark et al. (2015) [34] *n* = 24 (13)	Liver, pancreas, abd. aorta	24 *	16 or 40-MDCT vs. SSrs 64-MDCT	120 kVp, 100–600 or 250 mA, collim. 1.25 mm, 1.25/2.5 mm or 2.5/2.5 mm, 0.8 or 0.7 s, pitch 1.375:1 or 0.906; vs. 80/140 kVp, 600 mA, collim. 0.625 mm, 0.625/0.625 and 2.5/2.5 mm, 0.8 s, pitch 1.375:1/55.00, ASIR 40%; HAP (bolus track., aortic peak +15 s), PP (35 s), PVP (60–70 s), EP (180 s)	iohexol 350 or iopamidol 370, 104–200 mL or 98–200 (based on BW) vs. 70–133 or 55–90 mL (based on BW), rate adjusted for 30 s IT	1335 (562) vs. 1421 (564)
**9**	Ma et al. (2016) [40] *n* = 50 (28)	Portal v.	25 vs. 25	SSrs 64-MDCT	120 kVp, NI 10 vs. 80/140 kVp; ATCM, collim. 0.625 mm, 5.0/1.25 mm, 0.8 s, pitch 1.375:1, ASIR 50%, PVP (65 s)	Iopromide 370, 500 mgI/kg vs. 350 mgI/kg, rate adjusted for 25 s IT	13.1 (2.3) vs. 10.4 (4.0); 410.1 (141.9) vs. 387.5 (128.7)
**10**	Lv et al. (2017) [39] *n* = 160 (90)	Liver, portal v., abd. aorta	80 vs. 80	SSrs 64-MDCT	120 kVp, 101–480 mA, NI 10, 1.5/1.0 mm, 0.7 s, pitch 1.375:1 vs. 80/140 kVp, 260–600 mA, 5.0/1.25 mm, 0.5–1.0 s, pitch 1.375:1, ASIR 50%; AP (30 s), PVP (60 s)	Iohexol 350, 450 mgI/kg vs. 300 mgI/kg, rate based on BW	12.52 (4.85) vs. 11.95 (4.21); 354.53 (124.27) vs. 332.70 (119.67)
**11**	Li et al. (2018) [38] *n* = 62 (24)	Liver	31 vs. 31	SSrs 64-MDCT	120 kVp, 150–650 mA, NI 10, 0.7 s vs. 80/140 kVp, 260–640 mA, 0.5–1.0 s; 1.25/1.25 mm, pitch 1.375:1, ASIR 30% vs. 30% or 50%, AP (bolus track., 150 HU in abd. aorta +5.6 s), PVP (+30 s), EP (+300 s)	Iopamidol 370, 450 mgI/kg vs. Iodixanol 270, 270 mgI/kg, 4 mL/s	19.31 (4.11) vs. 11.27 (3.68) †; 528.49 (181.10) vs. 368.00 (104.83) †
**12**	Nagayama et al. (2018) [41] *n* = 90 (60)	Liver, abd. aorta	45 vs. 45	64-MDCT vs. SSdl 128-MDCT	120 kVp, 140–266 mA vs. 80–201 mA, collim. 0.625, 5.0/5.0 mm, 0.5 s, pitch 0.798, iDose 3 vs. Spectral level 3, HAP (bolus track., 150 HU in abd. aorta, +18 s), PVP (+55 s), EP (+160 s)	Iohexol 300 or iopamidol 370, 600 mgI/kg vs. 300 mgI/kg, rate adjusted for 30 s IT	13.4 (2.3) vs. 12.3 (2.3) †
**13**	Kim et al. (2019) [36] *n* = 94 (75)	Liver, portal v., pancreas, abd. aorta	94 *	DS 128-MDCT	100 kVp (ATVM), 180 mA, collim. 0.6 mm, 3.0 mm, 0.5 s, pitch 0.75 vs. 80/140 kVp, 230/89 mA, collim. 2 × 0.6 mm, 3.0/2.0 mm, 0.33 s, pitch 0.7, SAFIRE 2/5; HAP (bolus track., 100 HU in abd. aorta, +17 s), PVP (60–65 s), DP (180 s)	Iopromide 370 vs. 30%-diluted iopromide 370, 1.5 mL/kg, rate adjusted for 30 s IT	25.4 vs. 27.0 944.9 vs. 973.2
**14**	Han et al. (2019) [35] *n* = 41 (28)	Portal v.	21 vs. 20	SSrs 64-MDCT	120 kVp, NI 10, 0.6 s, pitch 1.375 vs. 80/140 kVp, 0.5 s, pitch 1.375:1; ATCM, collim. 0.625 mm, 1.25 mm, ASIR 40%, PVP (60 s)	Ioversol350, 0.6 gI/kg vs. 0.3 gI/kg, rate adjusted for 30 s IT	12.76 (4.83) vs. 14.47 (4.81); 395.05 (149.64) vs. 324.18 (101.41)
**15**	Shuman et al. (2019) [21] *n* = 62 (40)	Kidneys, urinary tract, renal art. and v.	31 vs. 31	SSrs 64-MDCT	120 kVp, ATCM (290–800 mA), NI 36, 0.5–0.8 s vs. 80/140 kVp, 500–640 mA, 0.5–1.0 s; collim. 0.625, 2.5/2.5 mm, pitch 1.375, ASIR 70%, NP (90 s), DP (10 min)	Iohexol 350, 125 mL, 3 mL/s vs. iodixanol 270, 81 mL, 2 mL/s	13.1 (6) vs. 14.7 (4)
**16**	Lennartz et al. (2020) [37] *n* = 78 (48)	Liver, portal v., pancreas, kidneys, abd. aorta	37 vs. 41	SSdl 128-MDCT	120 kVp, ATCM, collim. 0.625 mm, 2.0/2.0 mm, 0.33 s, pitch 0.7, PVP (bolus track., 150 HU in desc. aorta +50 s)	iohexol350, 100 mL vs. 50 mL, 3.5 mL/s	10.4 (2.4) vs. 10.3 (2.3)
**17**	Carrascosa et al. (2014) [43] *n* = 80 (56)	Thoraco-abd. aorta	20 vs. 20 vs. 20 vs. 20	SSrs 64-MDCT	120 kVp, 250–350 mA, 2.0/1.0 mm, ASIR 40% vs. 80/140 kVp, 250–375 mA; 2.0/1.0 mm, 0.350 s, bolus track.	Iobitridol 350, 60–100 mL (based on BMI), 4–4.5 mL/s vs. 50/40/30% of standard dose, 2.5–4 mL/s	N/A
**18**	Liu et al. (2016) [45] *n* = 127 (81)	Abd. aorta	58 vs. 69	SSrs 64-MDCT	120 kVp, ATCM, NI 10 vs. 80/140 kVp, 375 mA; 5.0/1.25 mm, pitch 1.2, ASIR50%, AP bolus track., 150 HU in abd. aorta +5.6 s)	Iohexol 350 vs. iodixanol 270, 100 mL, 5 mL/s	20.10 (4.99) vs. 10.76 (0.00) †; 882.93 290.71) vs. 573.58 (57.39) †
**19**	Agrawal et al. (2016) [42] *n* = 66 (52)	Abd. aorta	64 *	16 o 64-MDCT vs. SSrs 64-MDCT	120 kVp, ATCM, NI 15–30, collim. 0.625 mm, 1.5/1.0 mm, 0.5 s, pitch 1.375, ASIR30–60%, AP (bolus track.), DP (120 s) vs. 80/140 kVp, 600 mA, collim. 0.625 mm, 1.5/1.0 mm, 0.5 (<91 kg)/0.8 s (≥91 kg); pitch 1.375, AP (bolus track.), DP (70 s)	Iopamidol 370, 80 or 100 mL, 3.5 mL/s vs. iodixanol 270 or 320, 80–100 or 75 mL, 3 or 2.8 mL/s	14.4 (3) vs. 15.2 (2); 781 (237) vs. 814 (176)
**20**	Hou et al. (2017) [44] *n* = 120 (86)	Asc. and desc. aorta, celiac, renal and iliac art.	40 vs. 40 vs. 40	SSrs 64-MDCT	120 kVp, ATCM (max 600 mA), NI 12, ASIR 40% vs. 80/140 kVp, 360 mA, ASIR50%; 1.25/1.25 mm, 0.6 s, pitch 1.375:1, bolus track. (50 HU in asc. aorta, +0.6 s)	Iohexol 350, 70 mL, 5 mL/s vs. 0.6 or 0.4 mL/kg, rate = CV/(delay time + exposure time)	9.3 (2.8) vs. 7.4 vs. 7.4 †; 653.0 (219.1) vs. 505.8 (22.9) vs. 490.3 (26.3) †
**21**	Patino et al. (2019) [46] *n* = 52 (45)	Abd. aorta	52 vs. 26 vs. 26	16 or 64 or 128-MDCT vs. SSrs 64-MDCT	120 kVp, ATCM (75–550 mA, NI 15–18) or QRM 220 mA, collim. 0.625 mm, 2.5/2.5 or 2 mm, 0.5 s, 1.375/1, ASIR 30–50% or SAFIRE 3, bolus track. (80/100 HU in desc. aorta, +12 s [AP]), DP (120 s) vs. 80/140 kVp, fixed 550/630 mA, 2.5/2.5 mm, pitch 1.531, ASIR70%, AP (bolus track., 80/100 HU in desc. Aorta +12 s), DP (60 s)	Iopamidol 370, 80 mL (≤91 kg)/90 mL (>91 kg), 3.5 mL/s vs. iodixanol 270, 60 mL, 3 mL/s or iodixanol 320, 50 mL, 2.8 mL/s	12.8 (5.7) vs. 15.1 ± 2.2 ‡; 1114 (468) vs. 788 (166) †
**22**	Sugawara et al. (2019) [47] *n* = 21 (10)	Abd. aorta, celiac and sup. mesenteric art.	21 *	SSrs 64-MDCT	120 kVp, NI 12, 0.4 s vs. 80/140 kVp; ATCM, collimation 0.625 mm, 1.25/1.25 mm, pitch 1.375, ASIR40%, AP (40 s)	Iopamidol 300, 600 mgI/kg vs. 300 mgI/kg, rate adjusted for 30 s IT	9.84 (4.31) vs. 13.40 (4.58) ‡; 577.7 (279.6) vs. 920.0 (358.1) †

* In-patient comparison; † *p* < 0.01; ‡ *p* < 0.05; *n* = number of male patients. SSrs, single-source rapid switching; SSdl, single-source dual layer; DS, dual-source; MDCT, multidetector computed tomography; BMI, body mass index; ATCM, automated tube current modulation; ATVM, automated tube voltage modulation; NI, noise index; QRM, quality reference mAs; DRI, dose right index; ASIR, adaptive statistical iterative reconstruction; FBP, filtered back projection; SAFIRE, sinogram affirmed iterative reconstruction; AP, arterial phase; PP, pancreatic phase; HAP, hepatic arterial phase; PVP, portal venous phase; NP, nephrographic phase; DP, delayed phase; EP, equilibrium phase; CV, contrast volume; IT, injection time; DLP, dose length product; CTDIvol, volume CT dose index; N/A, not available.

**Table 4:** Result of qualitative image quality assessment for coronary arteries.

*Coronary Arteries*					
Author (year)	% iodine reduction	VMIs (keV)	CNR (SECT vs. DECT)	SNR (SECT vs. DECT)	Subjective image quality assessment (SECT vs. DECT)
Raju et al. (2014) [27]	56	60	16.9 (4.8) vs. 16.8 (5.2)	13.8 (3.9) vs. 12.0 (3.9)	5-point scale, good or excellent vs. moderate or good overall image quality †
Carrascosa et al. (2015)	50	60	18.0 (11.5) vs. 15.5 (9.6) ‡	14.5 (8.9) vs. 11.6 (7.1) †	5-point scale, good or excellent overall image quality
Oda et al. (2019) [28]	50	50	Asc. aorta 20.5 (5.0) vs. 29.3 (8.5) † LMA, 19.8 (4.8) vs. 27.2 (7.7) †; Proximal RCA, 19.6 (4.4) vs. 26.9 (6.6) †; Distal LAD 8.3 (4.6) vs. 23.9 (6.7) †; Distal LCX 18.4 (4.8) vs. 26.0 (9.9) †; Distal RCA 19.1 (4.1) vs. 25.8 (6.1) †	N/A	4-point scale, good or excellent overall image quality; not interfering or minimal or absent noise
Rotzinger et al. (2021) [29]	40	55	Lumen-fat 19.3 (11.6) vs. 24.9 (19.7) †; lumen-muscle 12.2 (8.5) vs. 14.3 (12.4) †; lumen-bone 6.8 (7.3) vs. 6.7 (8.9)	N/A	4-point scale, good or excellent overall image quality (average image quality score

† *p* > 0.01; ‡ *p* < 0.05. VMIs, virtual monoenergetic images; CNR, contrast to noise ratio; SNR, signal to noise ratio; DECT, dual energy CT; SECT, single energy CT; LMA, left main coronary artery; RCA, right coronary artery; LAD, left anterior descending artery; LCX, left circumflex artery; N/A, not available.

**Table 5:** Result of qualitative image quality assessment for pulmonary vessels.

*Pulmonary Arteries and Veins*					
Author (year)	% iodine reduction	VMIs (keV)	CNR (SECT vs. DECT)	SNR (SECT vs. DECT)	Subjective image quality assessment (SECT vs. DECT)
Yuan et al. (2012) [32]	50	50	12.2 (3.6) vs. 14.7 (6.5) ‡	14.5 (3.7) vs. 17.4 (7.1) ‡	5-point scale, excellent vs. limited or good overall image quality ‡
Dong et al. (2013) [31]	50	48–54	14.4 (6.4) vs. 19.2 (6.3) †	16.2 (6.5) vs. 21.3 (6.3) †	5-point scale, highest overall image quality and lowest noise for DECT †
Delesalle et al. (2013) [33]	30	60	Pulmonary art, 24.44 (6.7) vs. 13.90 (5.68) †; pulmonary v., 20.43 (6.5) vs. 13.20 (4.75) †; desc. aorta, 17.88 (4.7) vs. 11.97 (4.24) †	Pulmonary art, 27.95 (6.8) vs. 15.86 (6.0) †; pulmonary v., 23.94 (7.0) vs. 15.15 (5.25) †; desc. aorta, 21.39 (4.97) vs. 13.94 (4.77) †	3-point scale, absent or acceptable noise

† *p* > 0.01; ‡ *p* < 0.05. VMIs, virtual monoenergetic images; CNR, contrast to noise ratio; SNR, signal to noise ratio; DECT, dual-energy CT; SECT, single-energy CT.

**Table 6:** Result of qualitative image quality assessment for the abdomen and aorta.

*Abdomen*					
Author (year)	% iodine reduction	VMIs (keV)	CNR (SECT vs. DECT)	SNR (SECT vs. DECT)	Subjective image quality assessment (SECT vs. DECT)
Clark et al. (2015) [34]	37	52	Liver, 1.1 (0.8) vs. 0.70 (0.8); pancreas, 2.6 (1.9) vs. 2.3 (1.7); abd. aorta, 14.5 (5.8) vs. 13.4 (5.6)	N/A	5-point scale, acceptable or good overall image quality, higher or similar noise
Ma et al. 2016) [40]	25	60	Portal v., intrahepatic, 3.0 (2.1) vs. 4.2 (1.1) ‡; extrahepatic, 5.9 (1.6) vs. 5.9 (1.4)	N/A	5-point scale, good or excellent overall image quality
Lv et al. (2017) [39]	33	40	Liver, HAP, 1.0 (0.8) vs. 1.3 (1.2); PVP, 2.7 (1.9) vs. 4.5 (2.3) †; Portal v., PVP, 5.23 (3.4) vs. 10.2 (2.9) †; Abd. aorta, AP, 12.7 (4.4) vs. 21.2 (6.5) †	N/A	5-point scale, moderate overall image quality and noise
Li et al. (2018) [38]	41	52	Lesion-to-liver, HAP, 15.77 (5.93) vs. 19.51 (6.29) ‡; PVP, 8.19 (3.04) vs. 9.96 (2.18) ‡	N/A	4-point scale, moderate noise and above average diagnostic acceptability; better lesion conspicuity for 50 keV VMIs †
Nagayama et al. (2018) [41]	50	40	Liver, PVP, 6 vs. 10 †; EP, 3 vs. 5 †; Tumor-to-liver, HAP, 3.4 (1.2) vs. 8.3 (3.1) †; PVP, −1.9 (1.1) vs. −2.4 (2.0); EP, −2.1 (0.9) vs. −2.9 (1.7); abd. aorta, HAP, 22 vs. 55 †	N/A	5-point scale, average or above average vs. above average or excellent overall image quality †; moderate or minor noise
Kim et al. (2019) [36]	30	40	Hyper-enhancing lesion-to-liver, 1.11 (0.61–1.47) vs. 3.77 (3.11–5.02) †; hypo-enhancing lesion-to-liver 2.98 (1.12) vs. 2.72 (1.41)	Liver, HAP, 3.91 (0.74) vs. 3.08 (0.80) †; PVP, 6.66 (1.05) vs. 6.40 (1.21); Portal v., PVP, 10.99 (10.02–11.50) vs. 12.87 (2.04) †; pancreas, HAP, 7.20 (1.60) vs. 7.50 (1.80); PVP, 5.58 (0.90) vs. 5.88 (0.96); Abd. aorta, HAP, 20.24 (3.99) vs. 23.54 (4.74) †;	6-point scale, 50 keV VMIs superior to SECT
Han et al. (2019) [35]	50	50	Portal v., intrahepatic, 3.15 (1.29) vs. 3.16 (1.19); extrahepatic 6.83 (1.66) vs. 5.75 (2.28)	N/A	5-point scale, good or excellent
Shuman et al. (2019) [21]	50	50	Kidneys, NP, 21 (9) vs. 26 (8) ‡; renal art. and v.; NP, 13 (6) and 13 (8) vs. 13 (4) and 13 (5); calyces and pelvis, DP, 166 (112) vs. 255 (201) ‡; ureters 172 (96) vs. 195 (131); bladder, 113 (62) vs. 182 (141) ‡	N/A	4-point scale, moderate or good overall image quality and minor noise
Lennartz et al. (2020) [37]	50	40	Lymph nodes/Aorta, 15.2 (4.9) vs. 23.7 (8.9) ‡; Lymph nodes/Portal v., 17.5 (4.9) vs. 25.4 (9.0) ‡	Liver, 10.0 (3.0) vs. 9.1 (3.9); pancreas, 8.0 (2.3) vs. 8.3 (3.5); Portal v., 14.8 (4.2) vs. 16.9 (6.4); kidneys, 15.8 (4.8) vs. 17.7 (6.7); abd. aorta, 13.8 (4.0) vs. 16.1 (6.6)	4-point scale, excellent vs. proper overall image quality ‡; minimal vs. little noise ‡
*Aorta*					
Carrascosa et al. (2014) [43]	50 or 60 or 70	40	N/A	Thoraco-abd. aorta, 15.7 (8.7) vs. 15.3 (5.9) or 16.2 (8.3) or 14.1 (5.6)	10-point scale, very good quality and minimal noise; good quality with some noise for 70% reduction
Liu et al. (2016) [45]	23	65	Abd. aorta, 12.59 (2.64) vs. 16.14 (4.31) ‡	N/A	5-point scale
Agrawal et al. (2016) [42]	28	40	15.4 (6) vs. 19.3 (7.3) ‡	18 (6.6) vs. 21.1 (7.6) ‡	5-point scale, good overall image quality; minimal vs. moderate noise
Hou et al. (2017) [44]	40 or 59	60 or 55	Asc. aorta, 14.6 (2.7) vs. 24.3 (8.2) † or 16.8 (3.5); desc. aorta, 13.9 (2.9) vs. 22.8 (7.5) † or 17.0 (4.0); celiac art, 14.2 (3.0) vs. 22.1 (6.9) † or 16.0 (2.9); renal art, 14.5 (3.3) vs. 21.9 (6.5) † or 16.5 (3.3); iliac art, 14.3 (3.1) vs. 23.5 (7.0) † or 16.9 (3.5)	N/A	5-point scale, good overall image quality with low noise
Patino et al. (2019) [46]	52	40	Abd. Aorta, 18 (7) vs. 19 (5)	N/A	5-point scale, good or excellent overall image quality
Sugawara et al. (2019) [47]	50	52	Abd. aorta, 13.5 (2.6) vs. 16.8 ± 4.5 ‡; celiac art, 13.2 (2.7) vs. 16.3 ± 4.4 ‡; sup. mesenteric art, 13.3 (2.8) vs. 15.6 ± 4.0	N/A	4-point scale, completely visible

† *p* > 0.01; ‡ *p* < 0.05. VMIs, virtual monoenergetic images; CNR, contrast to noise ratio; SNR, signal to noise ratio; DECT, dual energy CT; SECT, single energy CT; HAP, hepatic arterial phase; PVP, portal venous phase; EP, equilibrium phase; NP, nephrographic phase; DP, delayed phase; N/A, not available.

## Data Availability

The datasets generated during the current study are available from the corresponding author on reasonable request.
